# Supplementary material for: SNAI2 cooperates with MEK1/2 and HDACs to suppress BIM- and BMF-dependent apoptosis in TERT promoter mutant cancers
Source: PLoS One. 2025 Jun 25;20(6):e0322961. doi: 10.1371/journal.pone.0322961 (PMC12193877; doi:10.1371/journal.pone.0322961)
Supplement: S1 File — Fig S1. ERK activation in TPM Cancer Cells is Inhibited by Low Doses of MEK1/2 inhibition (MEKi). Fig S2. BIM protein is below average in most TPM cancer types. Fig. S3 Combining MEKi and HDACi induces apoptosis in TPM cells. Table S1. Cell lines used in this study. Example gating strategy used for Flow cytometry. (PDF) [file pone.0322961.s001.pdf]

Begränsad delning

SUPPLEMENTAL DATA

Begränsad delning

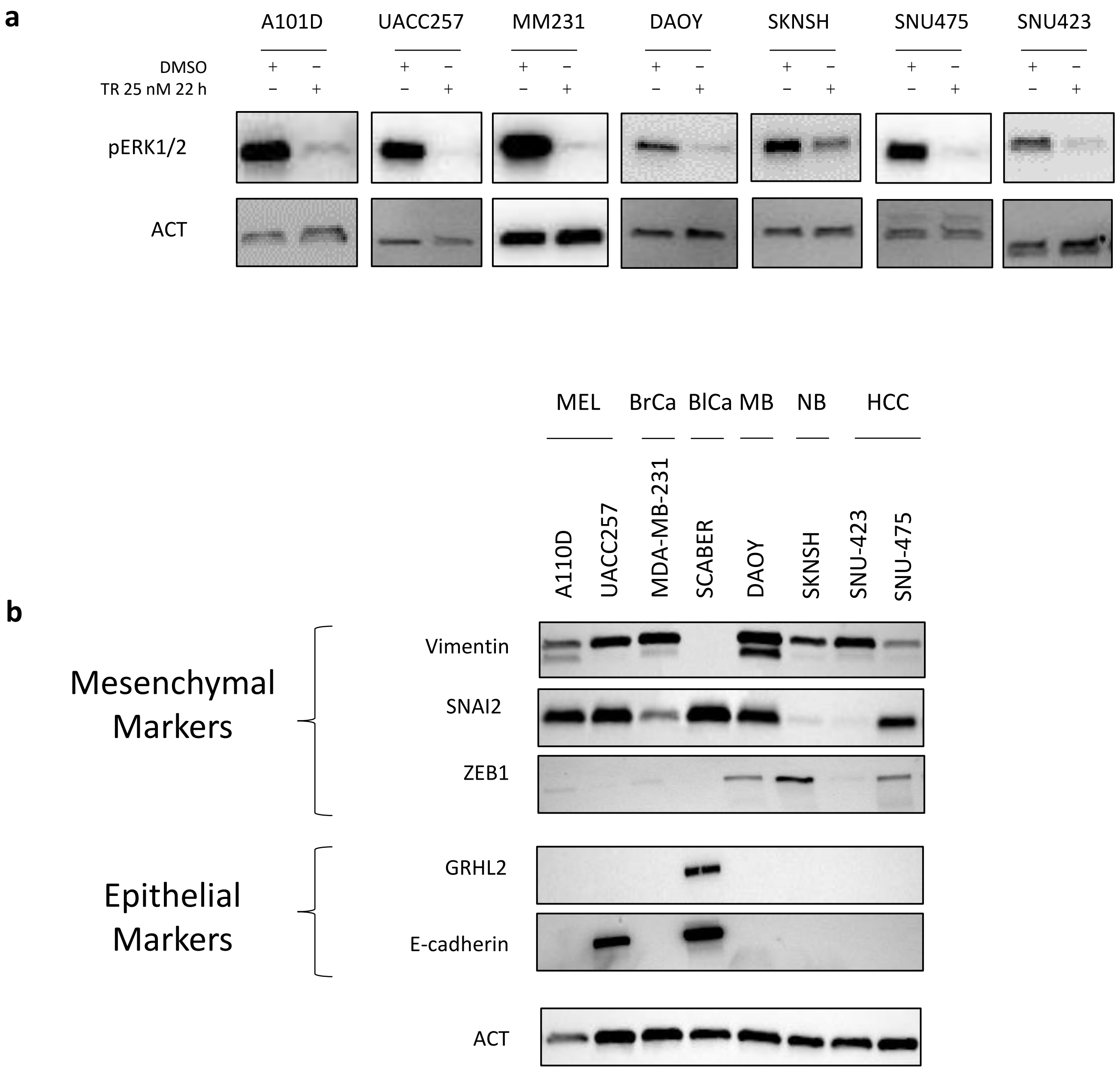

Fig S1. **ERK activation in TPM Cancer Cells is Inhibited by Low Doses of MEK1/2 inhibition (MEKi).** (a) MEK1/2 was inhibited in cells by treatment with trametinib (TR) 25 nM for 22 hours followed by immunoblot for phospho-ERK (pERK1/2, Thr202/Tyr204). (b) Mesenchymal (Vimentin, Slug and Zeb1) and epithelial (GRHL2 and E-cadherin) markers were tested in different cell lines by western blots. A10D and UACC257 (Melanoma), MDA-MB-231 (Breast cancer), SCaBER (Bladder cancer), DAOY (Medulloblastoma), SKNSH (Neuroblastoma), SNU423 and SNU475 (Hepatocellular carcinoma). β-Actin was used as loading control for Western blots. MEL – Melanoma; HCC – Hepatocellular carcinoma; BrCa – Breast cancer; BlCa – Bladder cancer; MB – Medulloblastoma; NB – Neuroblastoma.

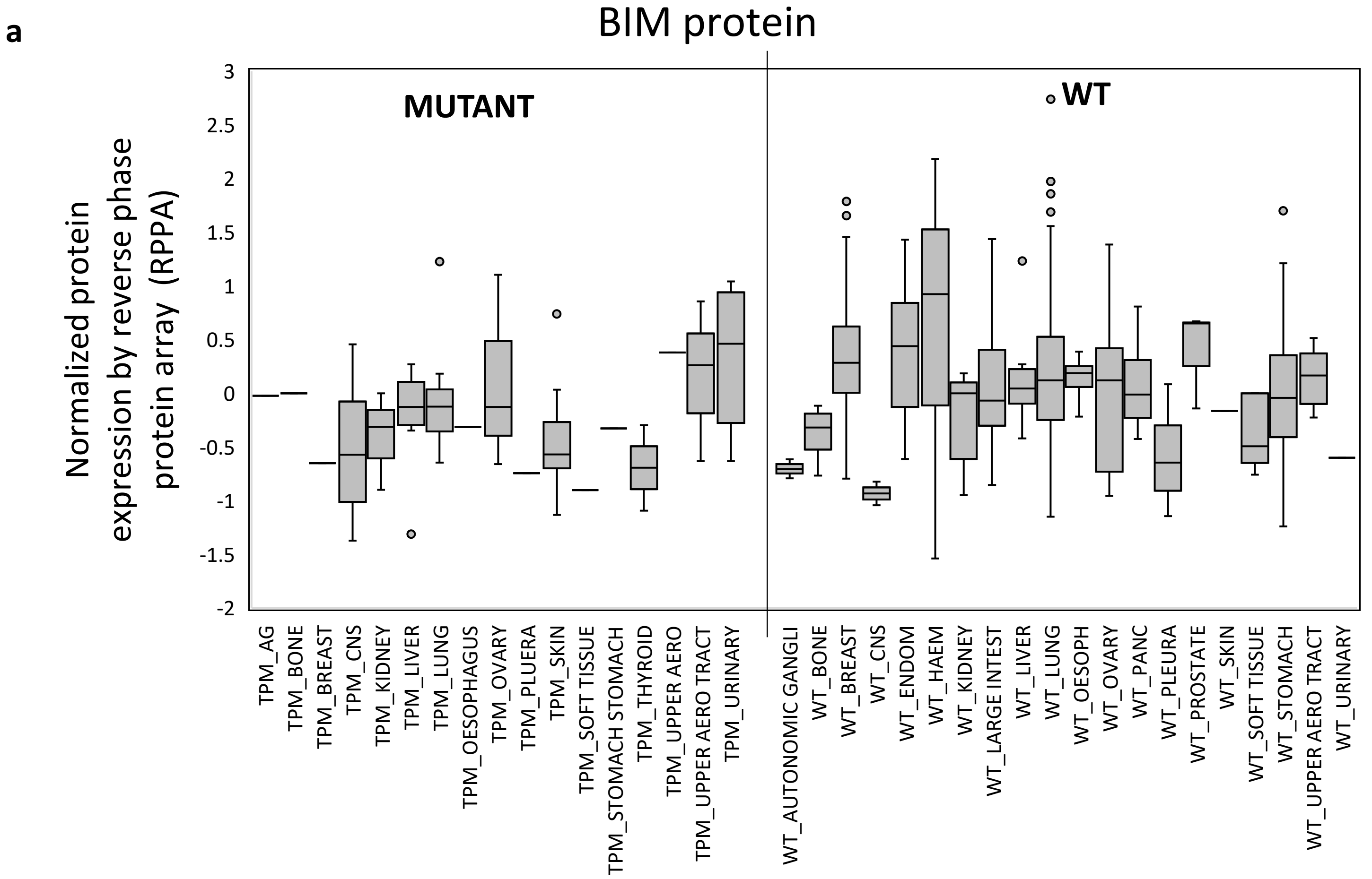

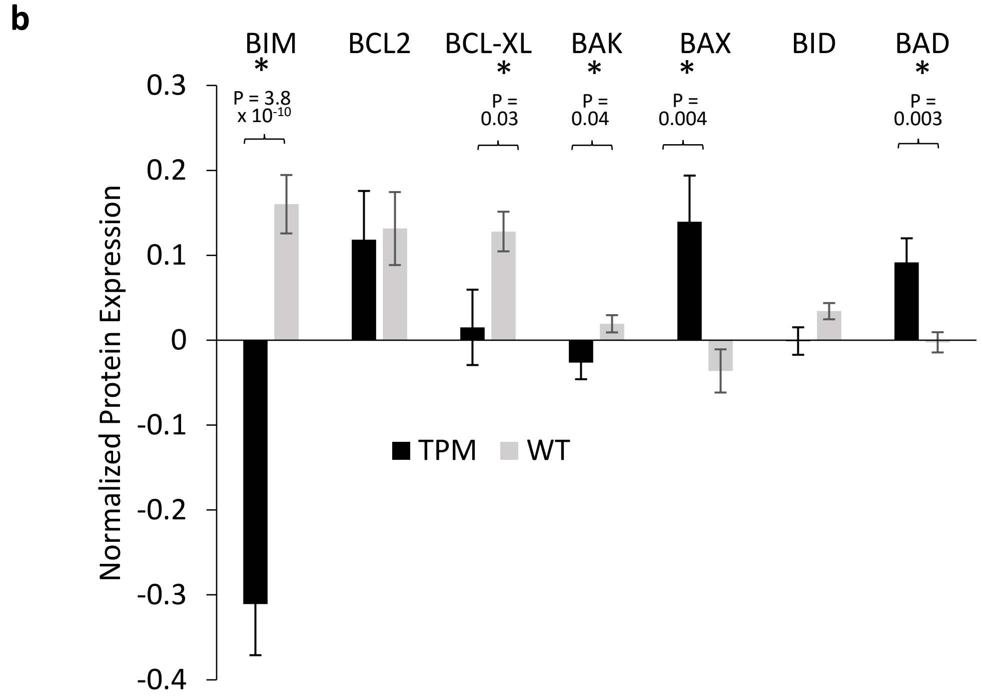

Fig S2. **BIM protein is below average in most TPM cancer types.** (a) Reverse phase protein array (RPPA) data was obtained from Cancer cell line encyclopedia (CCLE). Cell lines annotated as *TERT* promoter mutant or wild type (WT) were analyzed for BIM (*BCL2L11*) protein expression. Graph shows normalized data. (b) Data from RPPA depicting expression of BIM and other Bcl2-family member proteins in *TERT* promoter mutant (TPM, black) and wild type (WT, grey) cell lines.

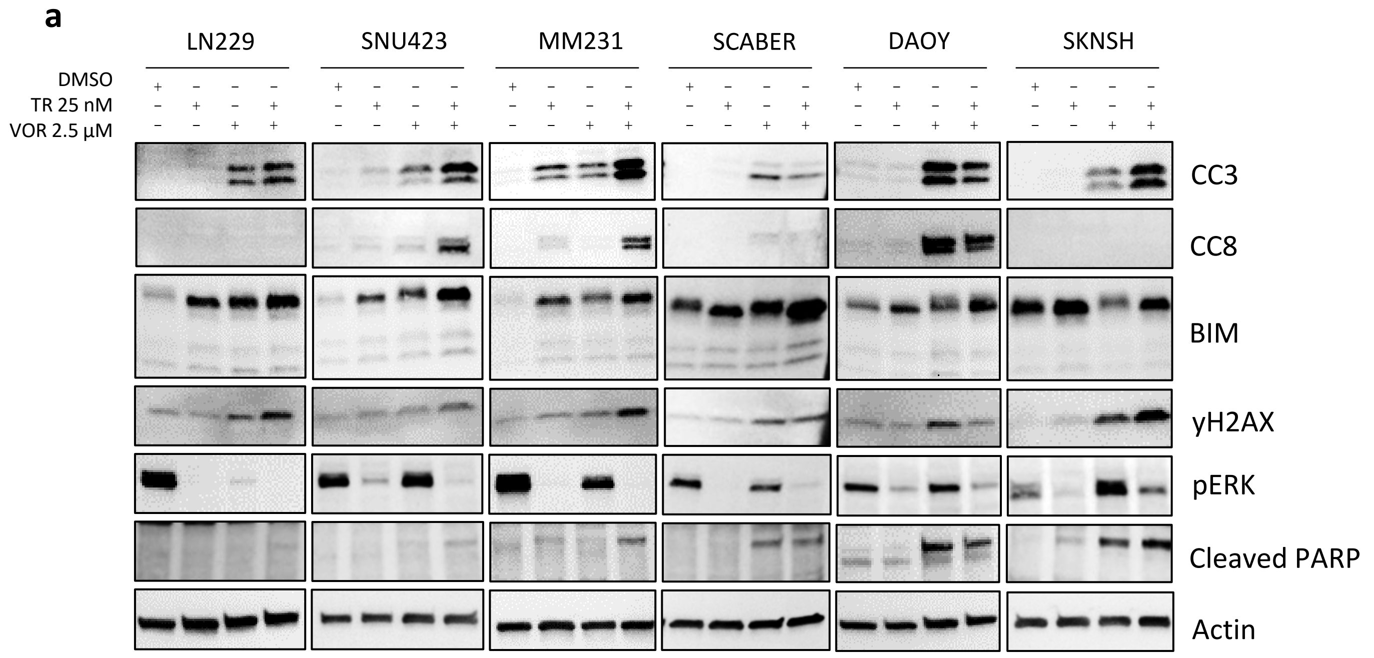

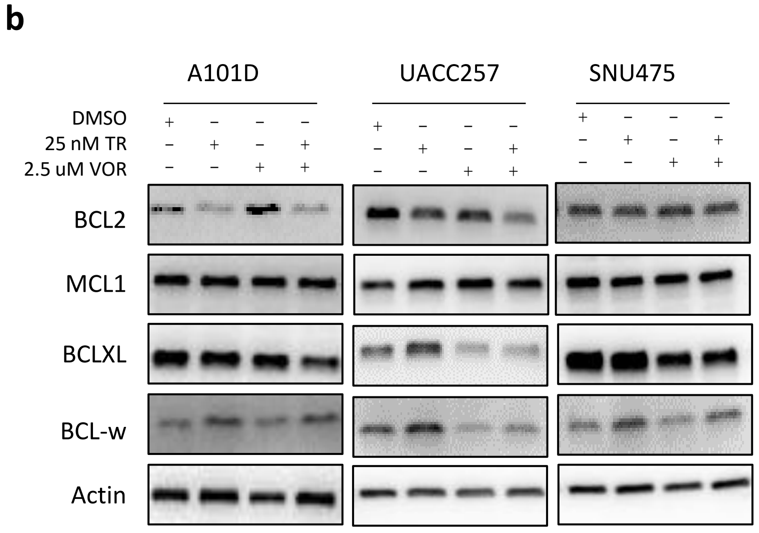

Fig. S3 **Combining MEKi and HDACi induces apoptosis in TPM cells.** (a) Cells were treated for 24 hours with trametinib (TR, 25 nM) with or without vorinostat (VOR, 2.5 µM) and probed for apoptosis (CC3) and induction of DNA damage (pH2AX) on Western blots. Blots were also probed for cleaved caspase-8 (CC8), phospho-ERK (pERK), BIM and cleaved PARP. (b) Cell lines treated in (a) were probed by immunoblots for pro-survival markers Bcl2, MCL1, BCL-XL and BCL-W. β-Actin was used as a loading control and for normalization.

Table S1.

GENE	A101D	UACC257	SNU-475	SNU-423	MDA-MB-231	DAOY	SK-N-SH	SCaBER
TERT	c.228C>T (-124C>T)* in promoter PMID: 31068700	c.250C>T (-146C>T)* in promoter PMID: 31068700	c.228C>T (-124C>T)* in promoter PMID: 31068700	c.228C>T (-124C>T)* in promoter PMID: 31068700	c.228C>T (-124C>T)* in promoter PMID: 31068700	c.228C>T (-124C>T)* in promoter PMID: 31068700	c.228C>T (-124C>T)* in promoter PMID: 26171145	c.228C>T (-124C>T)* in promoter PMID: 24035680 PMID: 31068700
**RAS pathway**	Heterozygous for BRAF	Heterozygous for BRAF			Heterozygous for BRAF Heterozygous for KRAS			
MGMT	MUT PMID: 30709805	WT PMID: 30709805						
**TP53**		Has no TP53 CCLE; Cosmic-CLP	MUT p.Asn239Asp (c.715A>G), p.Cys275Arg (c.823T>C) and p.Asn288Ser (c.863A>G) PMID: 8824565	MUT c.376-2A>G; splice acceptor mutation (PMID: 8824565; CCLE	Homozygous MUT p.Arg280Lys (c.839G>A) PMID: 15900046 PMID:16541312 PMID:17088437PMID: 18277095 PMID:28889351 ATCC			Homozygous MUT p.Arg110Leu (c.329G>T) PMID: 850064; CCLE; Cosmic-CLP
CDKN2A		CDKN2A deletion PMID: 29492214			Homozygous for CDKN2A deletion PMID: 19593635	Homozygous for CDKN2A deletion (ATCC).		
ALK							p.Phe1174Leu (c.3522C>A) ClinVar=VCV000217852) PMI: 18724359; PMID: 28350380.	

*-124 or -146 refers to base positions upstream of the *TERT* ATG. c228 of c250 refer to nucleotide positions in HG38 chromosome, 5: base 1,295,228 or base 1,295,250.

Table S1. **Cell lines used in this study.** Data from Cellosauraus (https://www.cellosaurus.org/) showing the mutational signature of different cell lines used in the study.

Begränsad delning

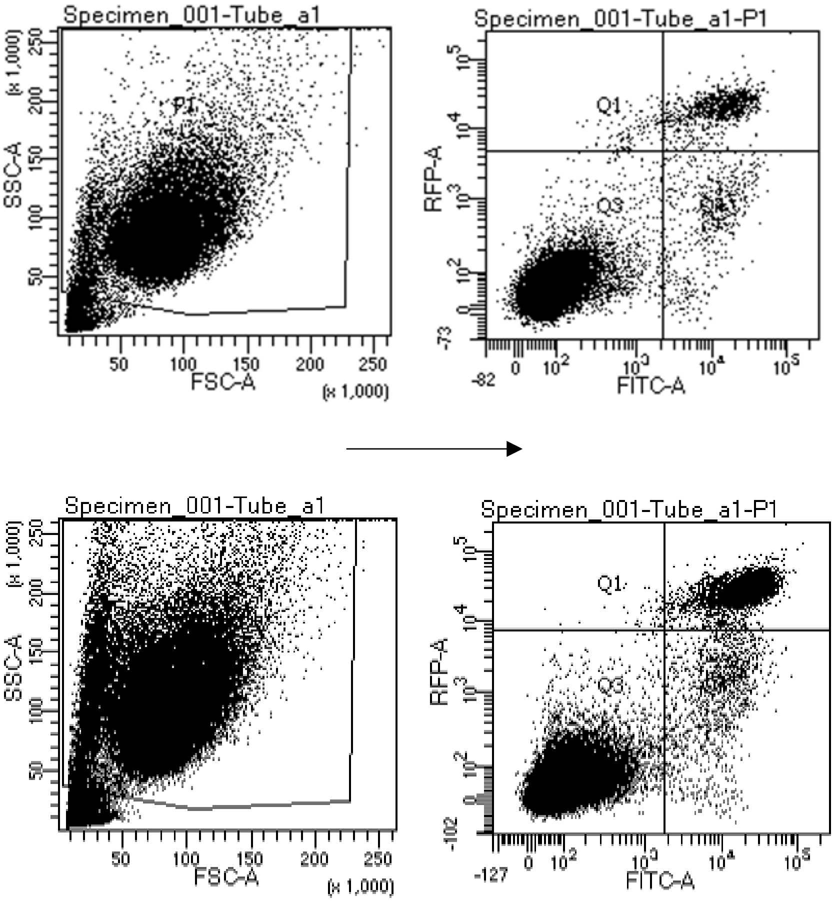

Example gating strategy used for Flow cytometry : gating strategy used was based on [forward and side scatter to exclude debris, doublet discrimination (not shown) and finally AnnexinV-FITC/PI staining. At least 10,000 events were recorded for each sample.
